# Implementing a Digital Depression Prevention Program in Australian Secondary Schools: Cross-Sectional Qualitative Study

**DOI:** 10.2196/42349

**Published:** 2023-06-12

**Authors:** Joanne R Beames, Aliza Werner-Seidler, Michael Hodgins, Lyndsay Brown, Hiroko Fujimoto, Alexandra Bartholomew, Kate Maston, Kit Huckvale, Isabel Zbukvic, Michelle Torok, Helen Christensen, Philip J Batterham, Alison L Calear, Raghu Lingam, Katherine M Boydell

**Affiliations:** 1 Black Dog Institute University of New South Wales Sydney, NSW Australia; 2 Population Child Health Clinical Research Group School of Women’s and Children’s Health University of New South Wales Sydney, NSW Australia; 3 Centre for Digital Transformation of Health University of Melbourne Melbourne, VIC Australia; 4 Orygen The National Centre of Excellence in Youth Mental Health University of Melbourne Parkville, VIC Australia; 5 Discipline of Psychiatry and Mental Health University of New South Wales Sydney, NSW Australia; 6 Centre for Mental Health Research Australian National University Canberra, ACT Australia

**Keywords:** implementation, youth, digital, depression, secondary school, qualitative, consolidated framework for implementation research, teacher, educator, perspective, mental health, student, child, adolescent, adolescence, school, social work

## Abstract

**Background:**

Depression is common during adolescence and is associated with adverse educational, employment, and health outcomes in later life. Digital programs are increasingly being implemented in schools to improve and protect adolescent mental health. Although digital depression prevention programs can be effective, there is limited knowledge about how contextual factors influence real-world delivery at scale in schools.

**Objective:**

The purpose of this study was to examine the contextual factors that influence the implementation of the Future Proofing Program (FPP) from the perspectives of school staff. The FPP is a 2-arm hybrid type 1 effectiveness-implementation trial evaluating whether depression can be prevented at scale in schools, using an evidence-based smartphone app delivered universally to year 8 students (13-14 years of age).

**Methods:**

Qualitative interviews were conducted with 23 staff from 20 schools in New South Wales, Australia, who assisted with the implementation of the FPP. The interviews were guided by our theory-driven logic model. Reflexive thematic analysis, using both deductive and inductive coding, was used to analyze responses.

**Results:**

Staff perceived the FPP as a novel (“innovative approach”) and appropriate way to address an unmet need within schools (“right place at the right time”). Active leadership and counselor involvement were critical for planning and engaging; teamwork, communication, and staff capacity were critical for execution (“ways of working within schools”). Low student engagement and staffing availability were identified as barriers for future adoption and implementation by schools (“reflecting on past experiences”).

**Conclusions:**

Four superordinate themes pertaining to the program, implementation processes, and implementation barriers were identified from qualitative responses by school staff. On the basis of our findings, we proposed a select set of recommendations for future implementation of digital prevention programs delivered at scale in schools. These recommendations were designed to facilitate an organizational change and help staff to implement digital mental health programs within their schools.

**International Registered Report Identifier (IRRID):**

RR2-10.1136/bmjopen-2020-042133

## Introduction

### Background

Meta-analytic findings estimate the pooled lifetime prevalence for major depressive disorders in adolescence to be 19% [1]. This lifetime prevalence estimate is concerning because adolescent depression is associated with serious social, educational, and health impairments across development [2,3]. The onset of depression is typically around mid- to late adolescence [4], yet many young people remain undiagnosed and untreated [5,6]. Of the young people who do receive evidence-based treatments for depression, relapse rates remain high and approximately 50% do not respond [7,8]. One reason for limited effectiveness is that treatment alone is not sufficient [9]. Prevention is also important, with estimates showing that psychological programs can reduce the incidence of depressive disorders in adolescents by up to 29% (compared with care-as-usual or control groups) [10]. Evidence-based depression prevention programs delivered at scale are crucial to address the depression burden and reduce the pressure on the mental health system. Scalability can be achieved by delivering evidence-based prevention programs in schools using digital approaches.

Schools are a critical touch point for the early identification of mental ill-health and the delivery of prevention programs for adolescents. Mental health support can be provided universally to all students in schools, which facilitates normalization of common emotional and behavioral experiences and overcomes barriers to help-seeking [11,12]. Among young people who do receive mental health care services, more than half first receive mental health care through their school [13]. School teachers and counselors perceive that supporting the mental health of their students is part of their role, and they are motivated to provide evidence-based programs during school time [14,15]. Given that depression in adolescence is associated with poorer academic performance [2,16] and school absence [17], prevention also aligns with the more traditional goals of educators and school administrators. Overall, partnering in the delivery of prevention programs is a natural fit for schools.

School-based depression prevention programs are effective. Meta-analyses have found that school-based prevention programs have a small preventive effect on depressive symptoms [18,19]. This preventive effect held regardless of whether the programs were delivered universally to all young people or were delivered to a targeted subsample with symptoms or risk factors [18]. An updated meta-analysis replicated these results and showed that 8 (7%) of the evaluated programs were digital (eg, delivered via a web platform through computers or laptops, or via smartphone apps) [20]. Digital programs were either supported by school staff (eg, classroom teachers) or by external facilitators in the classroom (eg, research assistants or trained psychologists). There was preliminary evidence that digital programs might be just as efficacious as face-to-face programs [20], which aligns with the pattern of results outside of the school setting [21]. These results demonstrate that staff-supported universal digital depression prevention programs may be an effective and feasible option for schools.

There are many practical benefits for delivering depression prevention programs digitally in schools. Digital delivery, including that via smartphones, is typically perceived by young people as an appropriate and accessible way to access mental health information and support [22,23]. Young people can progress through content at their own pace or potentially select modules that address their primary needs [24]. Teachers and counselors similarly perceive that using technology in schools to deliver mental health programs to students is appropriate and can integrate with existing ways of working [25]. Further, digital delivery is typically more cost-effective than face-to-face therapies [21] because it requires fewer resources to implement. Given that therapeutic content in digital programs is already developed, there is little (or indeed no) need for trained mental health facilitators. Despite the potential for maximizing reach, there is limited understanding about how to optimally deliver digital prevention programs at scale in schools.

Schools are complex environments for delivering digital mental health programs, and many factors will affect their implementation by staff and uptake by students. There has been a paucity of research to date that has evaluated the barriers and facilitators to implementing school-based digital mental health programs for students. One cross-sectional survey study from our research group addressed this gap by focusing on school staff perspectives. We evaluated staff perceptions about barriers and facilitators that would affect their implementation of a hypothetical digital depression prevention program in Australian schools [25]. Barriers included a lack of time and resources (ie, staff and rooms), concern about privacy issues in digital delivery, and a lack of clarity around staff roles and responsibilities [25]. Facilitators included upskilling staff through training; embedding the program into the curriculum; and other program factors including universal delivery, screening of students’ mental health, and clear referral pathways [25]. The extent to which these factors are important for real-world, scaled delivery of digital depression prevention programs in schools remains unclear.

In the context of clinical effectiveness trials, implementation evaluations aim to identify the contextual factors that influence how programs are put into practice “in the wild” (ie, hybrid type 1 designs) [26]. Such information is pivotal to understanding why programs work in some contexts and not in others because it can pinpoint sources of variation across different sites and individuals [27]. Implementation research in schools has primarily focused on evaluating the implementation of multicomponent physical activity interventions [28-32], mental health and well-being programs [33], or suicide prevention programs [34] delivered in person. Converging results show that there is variation in implementation processes across different schools, including the level of staff and institutional support, delineation of staff roles, and recruitment of other school-level resources [31,34]. Contextual factors, including cultural norms, location (eg, rurality), school-level buy-in, and program complexity, can also influence implementation [31,34]. The implication of these findings is that adaptation of implementation strategies by supporting staff may be essential for feasible delivery of school-based programs [31]. Different individual and contextual factors likely influence how school staff implement digital depression prevention programs.

### The Current Implementation Evaluation

The current implementation evaluation provides a qualitative analysis of the contextual factors that influence implementation of the Future Proofing Program (FPP) from the perspectives of school staff. The FPP is a 2-arm hybrid type 1 effectiveness-implementation trial evaluating whether depression can be prevented using an evidence-based smartphone app (SPARX [Smart, Positive, Active, Realistic, X-factor thoughts]) [35] delivered universally to year 8 students (13-14 years of age; see the trial protocol) [36]. Hybrid type 1 trials test the effects of an intervention on relevant outcomes while observing and gathering information about implementation [26]. Recruitment for the FPP trial occurred between August 2019 and March 2022. A total of 6388 students from 144 Australian schools in urban, regional, and rural areas enrolled and will participate in the trial until 2026. A total of 67 schools were allocated to the intervention condition, comprising 3266 students. The primary outcome of the FPP trial is the change in symptom severity of depression. Secondary outcomes include anxiety, psychological distress, and insomnia. Student-level outcomes are assessed at 6 weeks, 12 months, 24 months, 36 months, 48 months, and 5 years. Only the methods relevant to the current implementation evaluation will be described hereafter.

### Aim

The aim of this paper is to evaluate staff perceptions about (1) their involvement in supporting the FPP in their schools and (2) the extent to which contextual factors (eg, school organizational characteristics and staff characteristics) influenced their ability to implement the FPP. Findings will help to develop a practical guide outlining how to best deliver digital mental health programs to young people in schools.

## Methods

### Study Design

The evaluation was only conducted on the intervention arm of the study and is guided by the Consolidated Framework for Implementation Research (CFIR) [37]. The CFIR was used to identify barriers and facilitators to intervention implementation and effectiveness. The research team also developed a logic model that combines these frameworks to identify contextual factors hypothesized to impact the implementation processes and outcomes. For more details, see Figure S1 in Multimedia Appendix 1 and the implementation evaluation protocol [38].

### The Intervention

Students in the intervention arm of the trial received an evidence-based cognitive-behavioral therapy program for depression called SPARX [35,39]. The trial used a prevention version of the program (SPARX-R) that was delivered via a smartphone app. Skills learnt through SPARX-R include emotion identification, emotion regulation, behavioral activation (being active), recognizing and challenging unhelpful thoughts, and practical problem-solving. SPARX-R consists of seven 20-minute modules that are completed sequentially. The program is fully automated, and the therapeutic components are standardized. Students had access to SPARX-R for 6 weeks.

### The Implementation Strategy

An implementation strategy targeting school staff was developed to assist during the 6-week active intervention phase. The strategy involved (1) establishment of study implementation teams in schools (typically incorporating a classroom teacher and school counselor), (2) allocation of a minimum of four 20-minute school class sessions for SPARX-R completion, (3) provision of information about SPARX-R to schools by the research team, (4) weekly reminders by the school implementation team for students to use SPARX-R, (5) dissemination of information about the trial and mental health tips to schools by the research team, and (6) weekly liaison between the school implementation team and the research team to troubleshoot problems. The strategy, and alignment with recommended implementation strategies adapted for school contexts [40,41], is outlined in Table S1 in Multimedia Appendix 1. These outcomes were tracked by the research team although not formally recorded by schools because of feasibility constraints.

### School Visits

**Figure 1:**
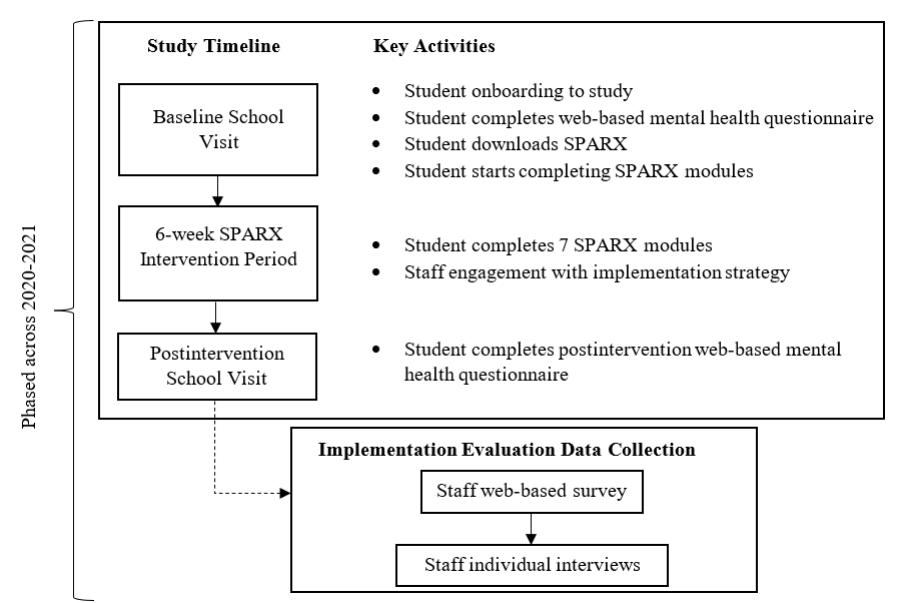
Study time line and flow of implementation evaluation data collection from school staff in the intervention arm of the trial. SPARX: Smart, Positive, Active, Realistic, X-factor thoughts.

Participating schools allocated to the intervention arm of the trial completed a baseline school visit, 6-week intervention period, and a postintervention school visit (see Figure 1 for a simplified timeline). The baseline visit served to orient enrolled students and supporting staff to the trial and encourage buy-in by providing information about what they were being asked to do and why. This visit also involved a web-based self-report mental health questionnaire for students. In the remaining time, students were encouraged to complete the first SPARX-R app module. Students were then given 6 weeks to complete SPARX-R. One to two modules were intended to be completed each week; however, students had autonomy to complete the modules in their own way (eg, all at once). As part of the implementation strategy, schools were encouraged to schedule sessions during school hours for students to complete the modules; this was not always feasible, meaning that some students only completed modules in their own time. Schools then hosted a postintervention visit whereby students completed a follow-up web-based mental health questionnaire.

### Data Collection and Sampling

This paper focuses on qualitative short-answer and interview data provided by school staff. These data were collected in 2 cohorts occurring between October-December 2020 and April-May 2021 from intervention schools only after the postintervention school visit (see Figure 1 for timings). Data from quantitative self-report surveys (eg, demographic and employment characteristics such as age, gender, and role) and the publicly available Myschool database are used to characterize the staff sample. Myschool contains data on every school in Australia; of relevance to this study were location, sector, Index of Community Socioeconomic Advantage, number of teaching staff, and number of enrolled students.

On the basis of the CFIR and FPP logic model, we developed a semistructured individual interview guide for school staff. The guide assessed the influence of contextual factors on implementation, including individual characteristics and school leadership, as well as implementation outcomes, including appropriateness and acceptability of the intervention (see “School Staff Interview Guides” in Multimedia Appendix 1). The interviews lasted between 20 and 60 minutes and were audio recorded. The recording failed for 3 interviews; in these instances, the interviewer took written notes during and after the interviews.

### Procedure

All school staff in the school implementation teams were invited via email to complete web-based surveys. After the provision of informed consent, web-based surveys were completed via Qualtrics [42]. Staff who completed the surveys were then invited to take part in optional interviews via phone or Zoom. As such, data were collected sequentially. Sequential methods enabled the research team to flexibly schedule interviews based on respondent availability, facilitating integration into their existing workflow and minimizing burden.

### Ethics Approval

Ethics approval was provided by the University of New South Wales Human Research Ethics Committee (HC180836) and New South Wales Government State Education Research Applications Process (2019201). For the implementation evaluation, staff from participating schools were asked to provide active informed consent to participate. Collected data were deidentified. For compensation of their time, staff received Aus $20 for completing the interview and Aus $10 for completing the survey.

### Data Preparation and Analysis

Descriptive summary statistics were calculated for quantitative data in SPSS (version 25; IBM Corp). Qualitative data were transcribed by 5 project personnel verbatim into Word (Microsoft Corp). Transcription was checked for accuracy by a second researcher who conducted all interviews. Four team members independently coded subsets of the data in pairs; all data were double coded. A benefit of having multiple coders is the capacity to deepen reflexive engagement with the data [43]. Coding discrepancies were discussed as part of the final thematic analysis.

Qualitative data were analyzed with NVivo 12 [44] using a modified form of Clarke and Braun’s [45,46] 6-stage reflexive thematic analysis guidelines. Reflexive thematic analysis enables the identification, interpretation, and reporting of meaningful patterns within data [47] and is advantageous due to its flexibility and rigor [48,49]. The coding team independently immersed themselves in the transcripts during the familiarization phase (phase 1), and then generated codes and themes using an inductive and deductive approach (phases 2 and 3). The team debated which codes and subcodes from the CFIR best represented the discourse from staff and what constituted appropriate and useful definitions for these codes. Coding pairs then independently coded their assigned transcripts (11-12 each), routinely meeting to discuss codes and meanings. The team reviewed the codes (phase 4) by examining examples and discussing whether the codes provided sufficient, adequate, and accurate representations of staff discourse. Phase 5 involved defining and naming themes. The first author integrated and creatively rationalized the codes, forming a narrative of themes and subthemes that was presented to the coding team and larger academic advisory group for review. Phase 6 involved producing the report.

### Rigor in Thematic Analysis

Rigor in thematic analysis was addressed by attending to established trustworthiness criteria [50,51]. Credibility was addressed by prolonged engagement with the subject matter, acknowledgment of the school context, and researcher triangulation. Transferability was addressed by providing thick descriptions (ie, situating experiences within a context) and documenting research steps. Dependability was addressed by describing and documenting each stage of the coding and theme development process. Reflexivity was addressed by engaging in regular team discussions, recursive engagement with the data, and acknowledgment of the teams’ role in knowledge production and interpretation.

## Results

### Recruitment and Completion Rates

**Figure 2:**
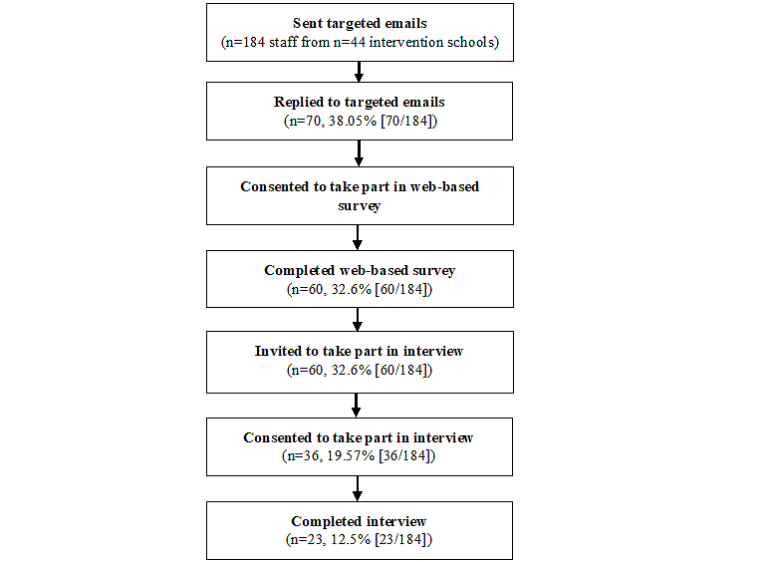
Absolute recruitment, consent, and completion rates for staff surveys and interviews.

A total of 184 staff from 44 intervention schools were sent targeted emails inviting them to take part in the implementation evaluation. Of the 184 staff, 70 (38%) replied to the expression of interest. Of the 70 who expressed interest, 68 (97%) consented to take part in the web-based survey, and of the 68 who provided consent, 60 (88%) completed the survey. Completion was defined as finishing ≥80% of the survey questions. These staff were employed at 36 different schools, representing a total coverage rate of 82% (36/44). Some schools had multiple respondents. Of the 60 staff who completed the web-based surveys, 36 (60%) consented to take part in follow-up interviews, and of the 36 who consented, 23 (64%) completed the interview. These staff were employed at 20 different schools, representing a total coverage rate of 45% (20/44; Figure 2).

### Staff Characteristics

Of the staff who completed the interviews, 39% (9/23) of them identified as a key leader who helped to drive and deliver the FPP in their school and 61% (14/23) identified as assisting with the delivery of the FPP in their school. The sample was primarily female (16/22, 73%) with a mean age of 40.9 (SD 11.82, range 24-60) years. The primary employment role of respondents was related to psychology or counseling (ie, psychologist, counselor, guidance or well-being officer; 14/22, 64%), and most respondents were employed full time (16/22, 73%). Approximately 60% (14/23) of the interviewed staff were from schools from the 2020 cohort, whereas approximately 40% (9/23) were from the 2021 cohort. Cohorts were conceptually similar in terms of key individual (ie, gender, age, number of years worked, and current role) and school characteristics (ie, sector, location, Index of Community Socioeconomic Advantage; *P*>.07). The schools that provided data for the implementation evaluation were representative of the schools taking part in the broader randomized controlled trial. See Table 1 for additional sample details.

**Table 1:** Characteristics of the staff sample that completed the interview (N=23).^a^

Sample characteristics		Statistics
Age (years), mean (SD)		40.09 (11.82)
**Gender, n (%)**		
	Female	16 (73)
	Male	6 (27)
**Aboriginal or Torres Strait Islander, n (%)**		
	Yes	1 (5)
	No	21 (96)
**Born in Australia, n (%)**		
	Yes	19 (86)
	No	3 (14)
**Highest level of education, n (%)**		
	Undergraduate degree	10 (46)
	Postgraduate degree	12 (55)
**Role in future proofing, n (%)**		
	Key leader who helped to drive and deliver the FPP^b^	9 (39)
	Assisted with delivery	14 (61)
**Employment role, n (%)**		
	Teacher or year adviser, head teacher	7 (31.8)
	Psychologist, counselor, well-being officer, or guidance counselor	14 (64)
	Principal or deputy	1 (5)
**If counselor, only counselor in school, n (%)**		
	Yes	2 (18)
	No	9 (82)
Total number of schools worked in, mean (SD)		8.36 (10)
Number of schools currently working in, mean (SD)		2.23 (3)
Number of years worked at current school, mean (SD)		6.64 (7)
Number of years worked in current role, mean (SD)		3.80 (3)
**Employment status, n (%)**		
	Full-time	16 (73)
	Part-time	6 (27)

^a^Cumulative percent reported. Characteristics from one respondent are missing due to user error.

^b^FPP: Future Proofing Program.

### Qualitative Themes

Four superordinate themes captured important factors about barriers and facilitators to implementing a digital mental health program in schools: (1) right place at the right time, (2) innovative approach, (3) ways of working within schools, and (4) reflecting on past experiences to improve future implementation. See Table S2 in Multimedia Appendix 1 for mapping of themes onto CFIR domains and example quotes.

#### Right Place at the Right Time

##### Overview

This theme reflects that schools were perceived by staff as critical touch points that can identify and respond to the needs of students at a time when they need it most. Staff confirmed that supporting the mental health of students was part of their role and described that schools were an appropriate place to deliver digital mental health programs. The main benefit of using schools was increased access and reach to all students. However, a consistent message from staff was the need to balance implementation of new programs with feasibility. Some staff reported that mental health was not an actionable priority in schools because of a lack of time or resources. Others noted that individual capacity, which fluctuates across the school year, can limit availability to support non–curriculum-based programs. Overall, staff reported that many programs are constantly being implemented in schools and are vying for space in an already congested curriculum. Two subthemes expand on the complexities of digital mental health programs in schools, which are discussed below.

##### A Blessing and a Curse

Staff reported that the COVID-19 pandemic emphasized the need for targeted mental health support for young people but that it also complicated program implementation for some schools. Delivery of the FPP was often delayed because of social distancing restrictions, school closures, and staff time constraints. In total, 56 schools withdrew their participation after March 2020 when the COVID-19 pandemic arrived in Australia. To accommodate government guidelines and school preferences, the school visits were redeveloped to allow remote and face-to-face sessions. Remote delivery of school visits worked well when staff were organized and proactively engaged students; face-to-face visits worked well in larger classrooms with students who had varying levels of support needs. Flexibility was a relative advantage of the FPP.

##### Addressing a Gap in the Community

Staff from rural or regional areas reported that the availability of youth mental health services in the community was low and that the FPP addressed an unmet need. Staff consistently described that the FPP provided an age-appropriate screening service (for suicide risk) and an evidence-based psychological program that would otherwise not be available. However, there were tensions about how screening would affect counselors’ capacity to support students and fulfill duty of care. Counselors worried that screening would identify at-risk students yet leave them with insufficient support options beyond the school.

#### Innovative Approach

##### Overview

Staff consistently intimated that the current reactive and piecemeal approach to student mental health in schools was not sufficient for a meaningful change. The FPP offered a new way of proactively identifying and addressing mental health needs in students, with no other programs like it offered in schools. Two subthemes exploring innovative features of the FPP were identified.

##### Screening

There was consensus that a universal preventative program with a screening component was appropriate for the needs of students and was compatible with the school context. Attitudes toward screening were generally positive, and clear advantages were noted for both staff and students. Counselors perceived that the confidential screening and risk alert system directly enhanced their ability to support students. The system enabled the identification of suicidal students who would have otherwise flown under the radar, enabling timely follow-up in a safe and secure setting.

##### Technology

Staff reported many benefits of using technology. The gamified app was perceived by staff as being more engaging for students than other school-based face-to-face programs. Leveraging students’ own devices was described as a positive because it was personalized, facilitated disclosure, and potentially motivated students to participate. Perceived appropriateness varied. Staff voiced strong opinions that a gamified app and standardized web-based survey might not be a good fit for all students. Staff identified that young people who have higher mental health risk, attention or behavioral difficulties, and low literacy levels might be less able to engage than their peers. Language and cultural diversity were also identified as challenges. Further, there were tensions about increased reliance on technology for educational purposes in schools in general. Staff commented on how technology changed teaching methods as well as the nature of student-student and teacher-student interactions. Some staff expressed concern about increasing screen time in schools and misalignment with existing school “no-phone” policies. Others reported that, with leadership support, using phones for a specific and time-limited purpose was acceptable.

#### Ways of Working Within Schools

##### Overview

Staff described what worked well and what did not work well in their schools, providing insights into how digital programs could be integrated into existing systems. Three subthemes were identified that explored issues relating to planning, engaging, and executing.

##### School Buy-in and Staff Ownership

The school executive was consistently described as being the primary decision maker in whether a school took part in the trial; yet they provided little practical support to FPP implementation. Staff reflected that greater input from leadership would have facilitated greater school-wide buy-in and ownership. Examples of input included formal recognition for staff involvement or allocation of time for planning and preparation. In addition to executive support, trust and reputation in the FPP provider was critical for buy-in among staff.

##### Bringing All the Right Players Onboard

There was staff consensus that involving the right people—those with the capacity, expertise, and interest or motivation to support student mental health—at the right time was essential for effective implementation. Internal (eg, values, interest, and altruism) and external motivations (eg, mandated role) to be involved in the trial were both prevalent, but the former was linked to higher engagement, commitment, and support in the long term. Individual capacity influenced who was best placed to be involved. Staff availability and competing demands often determined the amount of time they could dedicate to plan, engage, and execute the FPP. This was particularly the case for staff who described having to do extra work to engage parents and students. A consistent message from counselors was that, given their expertise and knowledge about other programs available in schools, they should have been critical decision makers for FPP adoption and central in planning and engagement. In practice, because of significant capacity issues in some schools, counselors were often not involved to this degree and had limited knowledge about the FPP and SPARX-R.

##### Communication and Support

The level of communication and practical support between staff varied across schools. Some staff reported a high level of quality communication and in-school support (eg, from the executive, head teachers, administrators, and counselors), describing a clear allocation of responsibilities and sharing of tasks. In these cases, communication and planning were generally organic and incorporated into routine meetings. Some staff reported having sole responsibility for supporting the FPP with minimal in-school support. Lack of communication and support was challenging in the context of already high workloads and pressing demands. For example, in some schools, counselors were not aware that a school visit had been planned until it began and they had to cancel standing appointments with students. Staff provided hypotheses for communication breakdowns including broader issues within school workplace culture and high staff turnover with a lack of handover.

##### School Visits and SPARX-R Sessions

School visits were generally described as being executed effectively. The level of support and quality of resources (eg, Wi-Fi and devices) provided by the research team were described as appropriate. Some staff described themselves as having an active and hands-on role in the school visits (eg, encouraging students to complete the survey and app modules, and managing behavior). The main difficulty for staff organizing the sessions was ensuring that the right students were in the room. Despite being encouraged in the trial protocol, few staff indicated that they scheduled separate school sessions dedicated to SPARX-R completion. The major barrier was extra resources needed for organization, planning, and execution (eg, time out of own schedule or curriculum).

##### Screening and Risk Alert System

Identifying high-risk students through screening increased demand on counselors when plans had not been established to manage caseloads. Counselors from some schools reported difficulty with following up students within a short time frame when more were identified than expected, no other counselors were available, or scheduling of FPP sessions did not align with their availability. In such cases, follow-up was described as reactive and often required counselors to cancel existing appointments to keep up with demand.

#### Reflecting on Past Experiences to Improve Future Implementation

##### Future Adoption

Reasons for future adoption focused on the benefits of the risk and referral process. Key benefits were related to the relative advantage of the FPP over other available programs for young people (eg, tech-based approach, tracking of mental health over time, facilitation of referrals, and normalization of the counseling service) and the tension for a change within schools to address mental health differently (eg, reactively vs proactively). However, there were barriers to future adoption. These barriers were related to individual capacity (eg, high workloads and limited availability), process factors (eg, poor communication or planning among staff members and disruptions to normal lessons), and innovation characteristics (eg, out of date gamification and graphics; note that SPARX was originally developed in 2012). The general sentiment among staff was that high student engagement is necessary to make the personal effort worthwhile. Maximum student sign on to the FPP (reach), and level of completion of the SPARX-R app (uptake) across the whole year was described as essential for school adoption. Many staff reported that student reach and SPARX-R engagement were lower than expected. The primary barriers for reach that staff identified were parent consent into the FPP and accessibility of technology and infrastructure in certain communities (eg, lower socioeconomic and rural or regional areas). Multiple hypotheses were raised for low parent consent, from poor communication of information to parents, parents being time-poor and having other higher priorities, mental health stigma, and low mental health literacy. Staff recognized that the limitations of consent are not a problem outside of research trials.

##### Ideal Implementation

Throughout their general reflections about how the FPP worked within schools, staff identified their “wish list” for ideal implementation. Integration into the school curriculum was consistently identified as necessary for buy-in and engagement because it has the potential to overcome barriers relating to resources, capacity, and coverage of students. Key facilitators for the implementation process identified by staff are presented in Table 2.

**Table 2:** Ideal facilitators for the implementation process of the Future Proofing Program (FPP).

Facilitators		FPP status^a^
**Planning and engaging**		
	Reputable implementing institution and provision of high-quality resources	Provided
	Active leadership support (including recognition of staff input) and school ownership	Encouraged
	Counselors involved in decisions about FPP adoption and planning	Encouraged
	In-school champion that engages staff and students	Encouraged
	Engagement strategy for students and parents to encourage buy-in	Provided
	Supportive school implementation team with clear responsibilities to share load	Encouraged
	Dedicated staff time for planning and reviewing	Encouraged
	Established community support for referrals of high-risk students that are outside the remit or resources of schools	Ideal
**Executing**		
	Flexible delivery of FPP	Provided
	Integration into school curriculum as an adjunct program	Ideal
	Technology support from implementing institution	Provided
	Adequate school-level resources (eg, rooms, Wi-Fi, and laptops)	Encouraged
	Standardized risk follow-up and referral processes for counselors	Encouraged
	Availability of counselors and provision of adequate time to respond to risk follow-ups	Encouraged
	In-school reminders about SPARX^b^ completion for students	Encouraged

^a^FPP status indicates whether the named facilitators were provided by the future proofing team, were encouraged by the future proofing team (and therefore adaptable based on school resourcing and preferences), or were outside of scope because of feasibility (and therefore ideal for future implementation).

^b^SPARX: Smart, Positive, Active, Realistic, X-factor thoughts.

## Discussion

### Principal Findings

This paper reports on the first implementation evaluation embedded into a school-based randomized controlled trial of a smartphone app depression prevention intervention. Four key superordinate themes were identified from qualitative responses provided by school staff involved in implementation delivery. Overall, the FPP added significant value to school approaches to mental health, offering vital resources that schools either did not have or were wanting to strengthen (right place at the right time). Staff agreed that an innovative approach was needed to support student mental health in schools and that a digital approach was generally appropriate within schools (innovative approach). Ease of execution reflected the amount and quality of planning staff engaged in, which was typically dependent on teamwork, communication, and individual capacity and motivation (ways of working within schools). Low student engagement and staffing availability were identified as critical barriers for future adoption and implementation (reflecting on past experiences). The identified themes highlight the importance of school organizational characteristics and staff characteristics in the implementation of digital mental health programs.

A critical point of disagreement in our results was capacity to respond to screening outcomes. Although there was tension for change in schools for new methods of identifying at-risk students and providing support in a timely manner, human resourcing was often a major barrier. Counselors from some schools also noted concerns about high identification rates in the absence of available community supports for referral. School counselors in Australia typically provide short-term reactive care rather than intensive tailored treatment programs for individual students [14,52]. Lack of available and appropriate referral options reflects a failure of the Australian youth mental health system [53] and leaves schools unsupported without the internal capacity to provide care. Our findings show that the implementation of mental health programs in schools, even when delivered digitally, requires careful planning and consideration of how schools intersect with the external health system.

### Organizational Change in Schools

An implication of our findings is that school-based implementation of digital mental health programs requires some level of organizational change. Organizational change in schools is any alteration, improvement, or restructuring in the processes or contents of education [54]. There are many organizational change theories, with different assumptions about why, how, and when a change occurs [55]. One perspective suggests the value of a continuous change, that is, ongoing, small-scale change that is embedded in daily practice [56]. Interpersonal relationships, leadership, and continual refinement are essential for this type of change in organizations [55]. The following sections will discuss these aspects of continuous change in schools in relation to the FPP.

#### Organizational Change in Schools: Interpersonal Relationships

Change at the school level requires change at the staff level. Prior research has shown that interpersonal process variables such as trust, social interaction, networking, communication, and knowledge sharing directly contribute to continuous change behavior in school teachers [55,56]. These findings align with this study. Working together, adequate communication about roles or tasks and sharing knowledge about the FPP were important for staff in the planning, engaging, and executing phases of implementation. These factors were particularly important for counselors. Some counselors were not ready or prepared to accommodate the new program, leading to significant disruptions to existing responsibilities and caseloads. Overall, working together to create new ways of doing things within the established school system was important to support implementation of the FPP.

#### Organizational Change in Schools: Leadership

The continuous change approach shifts leadership away from a top-level group (eg, the school executive) to every organizational member (eg, staff members involved in FPP implementation) [56]. This is not to say that the executive has no role; they are still integral in establishing a culture of change [55] and buy-in to the value system of an intervention. Our results showed that staff from most schools acknowledged that the executive and well-being units had cultivated, or were cultivating, a positive mental health culture and that the FPP aligned with this culture.

Distributing leadership practice across schools is needed to facilitate small-scale changes and make these changes a routine part of everyday work. Consistent with this conceptualization of leadership, our results showed the practical value of positioning counselors as critical decision makers in adoption, planning, and execution. Although these processes were encouraged in the FPP, interviews with school staff indicated that they were not put into practice in some schools despite willingness to do so (ie, nonadherence to the implementation strategy). Differences in school-level resourcing of counseling staff might in part explain the difficulties observed with engaging with the counseling unit. For example, counselors often work across multiple schools within a district and have limited availability in any one school. Involvement at critical decision points (eg, school-level adoption, when school visits will be held) may be necessary to ensure feasible involvement of counselors in future implementation efforts of digital mental health programs.

#### Organizational Change: Continual Refinement

A recent review of organizational change interventions in schools noted that many interventions fail to accomplish their purpose and result in a loss of valued resources [55]. Continuous change is dynamic, and even if change processes are operational, they often need to be refined and adapted to maintain their relevance within a system [55]. One implication, which was evident from staff perspectives about FPP, is that some level of flexibility in the implementation strategy may be necessary to help overcome challenges experienced during rollout of digital programs in schools. This was demonstrated by how schools complied with the strategy of scheduling in-school SPARX sessions to facilitate module completion. Although some staff were unable to do so because of limited staffing capacity and conflicts with other curriculum-based activities, other staff integrated SPARX completion into homeroom periods or scheduled specific SPARX sessions during class time. A flexible implementation strategy may be more feasible than a prescriptive strategy that does not allow adaptation. The need for local variation or tailoring of implementation processes is consistent with evaluations of other nondigital school-based physical activity and suicide prevention programs [31,34].

### Practical Recommendations: Implementing Digital Programs in Schools

Available implementation strategies tailored to school contexts have primarily been developed for programs delivered in person and are not specific to digital mental health or scaled prevention programs [40,41]. Drawing from the themes identified in our evaluation and related work [25], we outline recommendations for implementing digital mental health programs at scale in schools. The function of these recommendations is to facilitate organizational change and to help schools to achieve their intended purpose of implementation.

The top evidence-informed recommendation is alignment with and integration into the national school curriculum (eg, health or physical education). Integration would consolidate support from leadership and other staff, therefore facilitating appropriate allocation of resources and coverage of students. Not only does integration into the curriculum overcome time and resourcing barriers but it also aligns with the idea that mental health is a core part of the education and socioemotional development that is fostered by schools. This approach is a core tenet of the Health Promoting Schools framework, a whole-school approach to promoting health that recognizes the link between health and education [57,58].

In the absence of curriculum integration, additional school-level resources are necessary to facilitate buy-in and engagement from all sectors of the school community. We have developed eight evidence-informed recommendations about strategies that school staff can use to support the implementation of adjunct digital programs in schools:

1. Establishment of a multidisciplinary internal implementation team with counselors as critical decision makers in program adoption and planning. It is important to consider both the persons (eg, motivations) and their position (eg, formal responsibilities and capacity) to bring the right players on board.
2. A structured, multipronged approach to communicating information about the program, with a specific component for counselors, to encourage buy-in and engagement. Focusing on core business outcomes (ie, learning) as well as the links between mental health and academic achievement can clearly showcase benefits for schools, staff, students, and their families. School implementation teams may be best placed to communicate such information within schools.
3. Active internal leadership support for the program including allocation of resources (eg, in school time for planning, engaging, and executing), administration support (eg, to distribute reminders), and recognition of staff input and effort. In-school time for completing the program is essential; however, how this time is provided can be school dependent (eg, during roll call or homeroom and well-being days).
4. In-school champion or implementation lead that engages staff and students in the digital mental health program.
5. Formal meeting times held regularly between the implementing school staff to facilitate communication and review of implementation processes (using checklists to facilitate progress and accountability).
6. Prioritization of evidence-based programs that are engaging for students, with up-to-date graphics and gamification to ensure relative advantage over other programs in the marketplace.
7. Flexible program delivery to allow for adaptation to local contexts including area or region (metropolitan vs rural or regional, high vs low socioeconomic), school type (eg, public, independent, or catholic), or state or territory (eg, different policies and roles or availabilities of counselors). Flexibility is essential for scaled implementation across different types of schools.
8. Students use their own phone or laptop (or school-supplied devices if necessary) to complete the program.

### Limitations

Hybrid type 1 implementation effectiveness trials evaluating digital programs in schools have a unique set of challenges. One challenge is differentiating between implementation factors relating to the trial and to the digital program. For example, trial consent processes and other components of data collection related to secondary trial aims likely affected engagement and uptake of the FPP with students. Future hybrid trials should document which factors are research-related and program-related to guide hypothesis testing. In a deviation from the implementation evaluation protocol, we did not formally collect data from intervention schools about adherence to the implementation strategy due to feasibility constraints. The FPP team gained ad hoc information via emails from implementing staff about planning and execution. The data may not have been of sufficient quality to ensure reliability. As a result, objective data from all intervention schools about adherence to the implementation strategy are lacking, and we cannot conclude which strategies were crucial to mobilize staff support and student uptake of SPARX-R. Qualitative inquiry found that implementation teams in some schools did not do what was specified or encouraged. This provides some indication that the strategies were not feasible for all schools, reinforcing the complexity of implementation in schools. Finally, the CFIR covers multiple perspectives. The current evaluation explored reports from a small sample of school staff. Different insights into implementation barriers and facilitators may have been raised by students, parents, education departments, or even community services linked in with schools.

### Conclusions

Results from this study showed that interpersonal factors, leadership factors, and flexibility were important for implementing the FPP in schools (according to staff). We also found that implementing digital programs in schools requires significant planning and consideration of specific school environments. In line with our findings, we proposed a select set of recommendations for future implementation of digital prevention programs delivered at scale in schools. These recommendations might facilitate an organizational change and help schools to achieve their intended purpose of implementing digital mental health programs (eg, improved student support and mental health). These recommendations can also be applied beyond the mental health domain to support the scaled implementation of other digital programs in schools.

## Appendix

Multimedia Appendix 1 School Staff Interview Guides.

## Abbreviations

CFIR: Consolidated Framework for Implementation Research
FPP: Future Proofing Program
SPARX: Smart, Positive, Active, Realistic, X-factor thoughts

## Data Availability

Data generated or analyzed during this study are not available because data are sensitive and sharing does not comply with ethical approvals.
