# Supplementary material for: Implementing a Digital Depression Prevention Program in Australian Secondary Schools: Cross-Sectional Qualitative Study
Source: JMIR Pediatr Parent. 2023 Jun 12;6:e42349. doi: 10.2196/42349 (PMC10337254; doi:10.2196/42349)
Supplement: Multimedia Appendix 1 [file pediatrics_v6i1e42349_app1.docx]

**Multimedia Appendix 1**

**School Staff Interview Guides**

INTRODUCTION:

*Thank you for agreeing to take part in this interview.* *I appreciate your time.*

*I am interested in hearing your thoughts and experiences of the Future Proofing program in your school. For this interview, the FP program includes the intervention app (SPARX) and everything else that was done to support the program in your school. I hope to use this information to work out what works in different schools and how to best provide support.*

*I will ask you a few questions about your experiences. This is chance for you to reflect and offer feedback on how we could improve. The interview will last up to one hour. It will be recorded, and your responses will be transcribed to ensure we accurately reflect your words. All personal information will be removed so that your answers are non-identifiable and confidential. Nothing you personally say will be shared with anyone on the FP team that you have been liaising with or with other staff at your school. You can choose to not answer any questions.*

*Do you have any questions before we get started?*

OPENING QUESTIONS

1. Overall, how would you describe your experience with the FP program in your school?
2. What was your role in delivering or supporting the FP program in your school?
   1. Leader/driver, decision-maker, consent/recruitment, peripheral support
3. How did it fit with the core values of your school?
4. What other mental health programs or initiatives has your school implemented to care for students besides the FP program?

CFIR DOMAIN: INDIVIDUAL CHARACTERISTICS

*Personal attributes (e.g., motivations, expectations, capacity, values)*

1. How did you come on board with/hear about the program?
2. Why were you motivated to take on this role?
   1. [Theory of Planned Behaviour: Beliefs]
      1. What do you think are the advantages of the program? (altruism, intellectual curiosity/self-development)
      2. What about disadvantages? (inconvenience)
   2. [Theory of Planned Behaviour: Control]
      1. What factors would encourage you/make it easier to take on this role?
      2. What would discourage/make it harder?
3. Why do you think your school decided to support this program?
   1. To what extent do you think there was a need for a mental health program like FP in you school (*Tension for change)*?

*Knowledge and beliefs about the intervention*

1. How much do you know about SPARX, the intervention? E.g., principles, rationale, content?

*Self-efficacy*

1. How confident were you in your ability to lead/support/make decisions about the FP program?
   1. What personal qualities do you think helped you to do that?

CFIR DOMAIN: OUTER SETTING

*Relative Advantage*

1. How did SPARX compare to other mental health programs that your school has supported or delivered?

CFIR DOMAIN: INNER SETTING

*School contextual factors – Barriers and Facilitators*

1. What factors influenced your ability to deliver/support the FP program in your school? [query online or FTF delivery]
   1. What else helped or hindered your ability?
      1. Enquire into school-specific factors and program-specific factors e.g., What parts of the program were easy for you to do or support (e.g., the consent process, organising rooms, weekly team meetings)? OR What aspects of your school made it difficult for you to support the program?
   2. What factors got in the way of students completing the mental health app/s?
      1. Were you (or others) able to overcome any barriers? How did you manage to do this?
   3. How well supported were you by the research team?
2. What is essential for a program like this to be effective in schools?

*Forces of change (organisational change theory)*

1. What were the driving factors underpinning why your school implemented FP? (poor student mental health, low attendance/drop out, lack of services in the community, interest/attitudes from staff)
2. What resistance was there for the program? (individual and school level)
   1. Uncertainty about what it would involve (how their work and lives will be affected by the proposed change)
   2. Concern over personal loss – (the cost of change in terms of quality of work, inherent characteristics of the job)
3. Group resistance (norms about MH programs in school – imposed by principal without consultation)
4. Trust in administration (Principal/executive – if they made decision)
5. Awareness of weakness in proposed change
6. How can we overcome resistance to change? (education/training, clear communication, rewards for role)

*School contextual factors – Leadership*

1. Tell me about the involvement of your school executive in the FP program.
   1. What role did this person have (e.g., principal/vice principal)? Did you meet regularly with this person or provide updates to them?
2. How do you think the school executive saw this initiative?
   1. In what way were they supportive?

*Readiness for implementation*

1. How would you describe the readiness of your school to take on the FP program?
   1. What was the preparation phase like for you/other key staff?

IMPLEMENTATION METRICS

*Appropriateness and Acceptability*

1. How does the FP program align with your school’s approach toward student mental health?
2. How suited is the FP program to address depression prevention in your school?
3. How well does an app delivering therapy work to prevent depression in your students?
4. How do you feel about using technology to deliver psychological interventions in your school?
   1. What worked well about this and what did not?
   2. Do you think SPARX met the student’s mental health needs? In what way?
5. What were the by-products of the intervention that you didn’t expect?
   1. What parts of the program do/do not suit your school?

**ADDITIONAL QUESTIONS FOR SCHOOL COUNSELLORS ONLY

1. What was your experience with responding to students identified as needing follow-up during the FP program?
   1. Was there anything that could have been done to make this experience better for you and the student?
2. What do you think of the FP risk protocol?

KEY LEARNING AND FUTURE IMPLEMENTATION

1. What parts of the FP program, if any, could be made more attractive to your school to increase uptake or support?
   1. In what ways could we make these parts more attractive?
2. To what extent do you think that the FP program could be integrated into your school’s existing mental health strategy?

*Closing*

1. Is there anything else you would like to add?


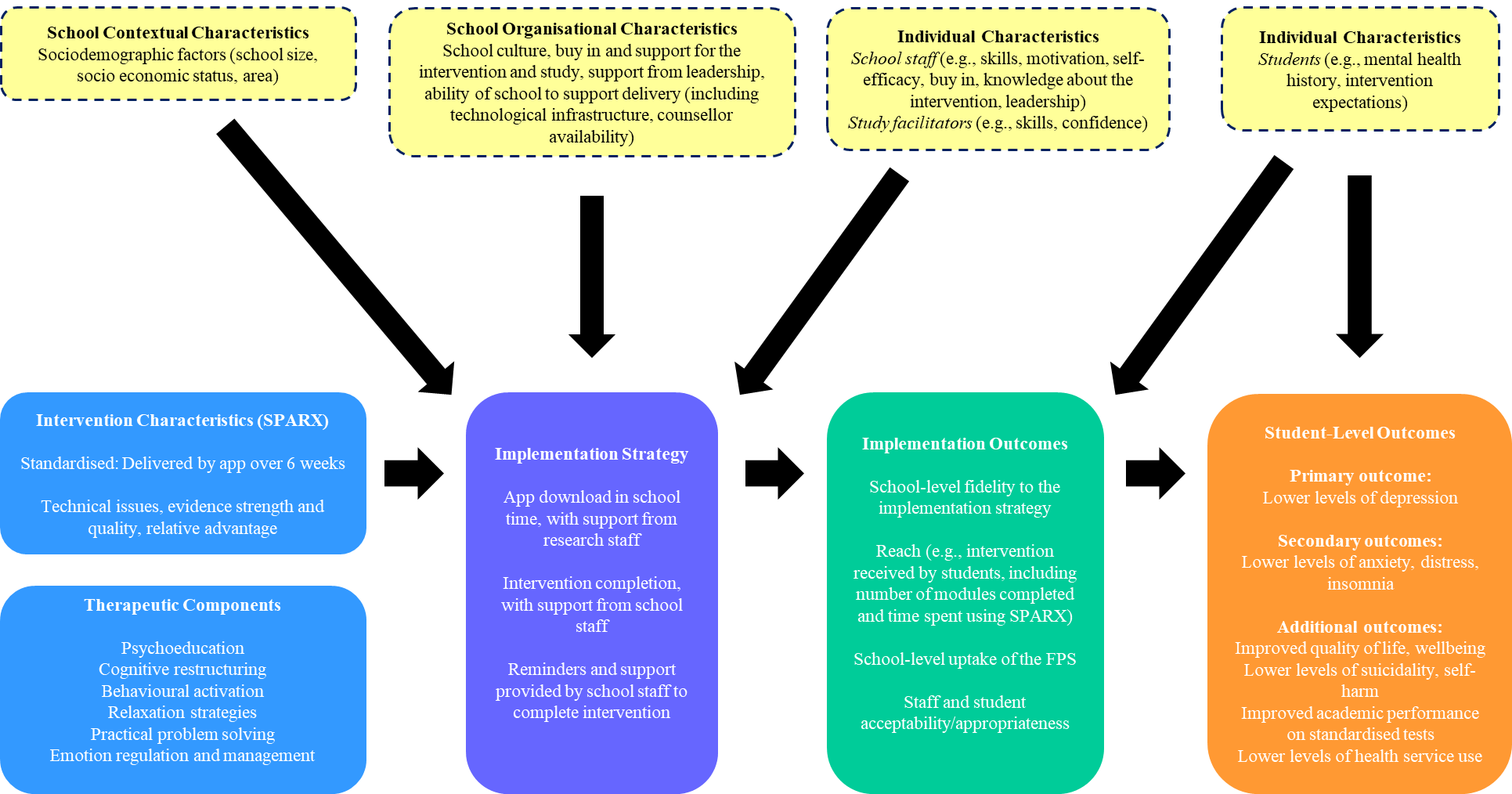
Figure S1. Logic model showing that CFIR constructs, including school context characteristics, school organisational characteristics and individual characteristics, will influence how staff engage with the implementation strategy.

*Note.* This image is taken from the protocol paper (see reference below), with permission from the authors and is reused in line with the original author license (CC BY-NC 4.0).

Beames JR, Lingam R, Boydell K, Calear AL, Torok M, Maston K, et al. Protocol for the process evaluation of a complex intervention delivered in schools to prevent adolescent depression: The future proofing study. BMJ Open. 2020;11(1):e042133. doi: 10.1136/bmjopen-2020-042133.

Table S1. School implementation strategy and mapping onto SISTER strategies.

| Implementation Strategy | Key SISTER Strategies |
| --- | --- |
| (1) Establishment of study implementation teams responsible for the implementation of the intervention and liaising with the research team. These teams typically incorporated at least one classroom teacher and one school counsellor, and were responsible for mobilising the FPP, engaging students, and encouraging buy-in from other staff. Schools had discretion about how the individuals were selected/nominated and worked together. This might also include identifying one individual dedicated to supporting and driving the FPP, overcoming any barriers that the FPP might provoke in a school. | A. Create new practice teams  B. Identify a champion who  facilitates buy-in and engagement to the FPP  C. Promote adaptability |
| (2) Allocation of a minimum of 4 x 20-minute school class sessions during which students complete the SPARX intervention (the remaining sessions could be completed in class if permitted by schools or in students’ own time). Schools were required to sign a contract and commit to scheduling at least 4 in-class SPARX sessions; however, given feasibility constraints, schools were not excluded if they were unable to put these into practice. | A. Alter aspects of the environment (i.e., master scheduling of sessions, repurposing of space) to formalise time for FPP completion  B. Develop local school system policy that establishes expectations and guidelines for in-school support  C. Mandate for change whereby leadership declare the priority of the FPP and provision of staff support  D. Promote adaptability |
| (3) Provision of information about SPARX from the research team for school visits. | A. Distribute educational materials |
| (4) Weekly verbal reminders from the implementation teams to students to use the app. | A. Communicate with students to enhance uptake and completion  B. Improve students’ buy-in |
| (5) Dissemination of information about the study and mental health tips in the school weekly newsletter by the research team. | A. Communicate with students, families, and other staff to enhance uptake and completion  B. Use mass media to reach many students and families  C. Distribute educational materials  D. Improve implementers’ buy-in |
| (6) Weekly liaison with the research team to troubleshoot problems. | A. Remind school personnel  B. Facilitation/problem solving |

Table S2. Mapping of superordinate and sub-themes onto CFIR/RE-AIM domains, as well as example quotes from respondents.

| **Superordinate Themes and Sub-Themes** | **CFIR Mapping** | **Definition** | **Example Quotes [Data Source and Respondent]** |
| --- | --- | --- | --- |
| *1. Right place at the right time* | Inner setting: Compatibility, relative priority, and tension for change | Mental health of young people recognised as a critical need for further targeted intervention, with schools identified as an ideal touchpoint to deliver programs to all young people. | *“…probably general concern over students’ mental health. We do have quite a number of students that do have mental health difficulties whether diagnosed or undiagnosed, that are of high level of concern within the school so yeah, I would say that that would have helped in terms of the principal agreeing to do it.”* [Interview, school counsellor/psychologist, female] |
|  |  |  | *“I guess it’s definitely something good to do in year, I would say year 8, it’s definitely… or year 7 and 8 [yeah] just because you know getting in early and then they know about the referral process to the counsellors and you’re kind of giving them all this language early [uhih] rather than later [uhih]. Like it’s just proactive. It just makes, it’s mind-boggling sense.”* [Interview, school counsellor/psychologist, male] |
| *i. A blessing and a curse* | Inner setting, outer setting, innovation characteristics: relative priority, COVID-19, and relative advantage | COVID-19 emphasised need for targeted mental health support for young people, but also complicated implementation for some schools. Flexible delivery of Future Proofing was an advantage in this context. | *“I think that’s one thing we’ve kind of seen in our community at school, is that a lot of the parents are crying out for more information on this and I think… you know… not because of Covid but Covid has probably highlighted the epidemic that’s kinda [sic] facing Australia… I guess… with regards to mental health in young people, especially with boys, we’re at a boys’ school”* [Interview, year advisor/head teacher, male] |
|  |  |  | *“…because there was the Covid stuff sort of creeping up, um, it all felt… very… like too overwhelming at one point [uhih] and nothing extra that we sort of planned was going to be running.”* [Interview, school counsellor/psychologist, female] |
|  |  |  | *“I think we would have had more buy-in probably… if we didn’t have Covid”* [Interview, year advisor/head teacher, female] |
| *ii. Addressing a gap in the*  *community* | Outer setting: availability of appropriate mental health services | Future Proofing provided a youth appropriate program that filled a gap in communities were mental health services/programs were not available or were at capacity. | *“We are really strapped for any sort of services for kids really in Tamworth. We do have a headspace but the waiting list for them, I think it's up to about 10 months.”* [Interview, school counsellor/psychologist, male] |
|  |  |  | *“There isn’t anything else. That’s it. It’s (sic), it’s like we’ve literally got Headspace which is free service and um… and me.”* [Interview, school counsellor/psychologist, male] |
| *2. Innovative approach* | Inner setting, innovation characteristics, individual characteristics: Compatibility, relative priority, relative advantage, appropriateness/ acceptability, quality of support and resources, attitudes toward the intervention | Universal preventative program with screening component delivered through technology was appropriate for the needs of students. It was also suited to the needs of the school – new idea, no other programs like it – with advantages for students and staff (e.g., allowed counsellors to do their job more effectively). | *“…but definitely something which is proactive, captures all kids at one age group provides a lot of wellbeing information could definitely become part of I guess more of a foundational aspect of wellbeing program for year 8 students.”* [Interview, school counsellor/psychologist, male] |
| *i. Screening* |  |  | *“…whereas when you do proactive one like this, you’re able to pick up a bunch of kids who are too scared or don’t say anything because they’re talking to a computer.”* [Interview, school counsellor/psychologist, male] |
|  |  |  | *“I think I like the idea that it’s app based as well because it’s more engaging for young people than just having some adult [Uhih], you know up in the room lecturing them or something or other. We have had a few things. Um, we had the community health team come in and do some work with the students, um, for a couple of days for each year group [Uhih]. But that’s kind of more classroom based, more didactic in style. So, yeah, I felt like an app-based program was something different [Uhih]. I think that might be more engaging for students.”* [Interview, school counsellor/psychologist, female] |
|  |  |  | *“…there was positives in being to be able pick up the kids who kind of often fall through the cracks that we are not aware of as well and um I think that was really valuable.”* [Interview, school counsellor/psychologist, female] |
| *i. Technology* |  |  | *“…the kids that did join in were probably more of our… more like conscientious students [mmhmm], so they were like available and they had what they needed to bring equipment-wise.”* [Interview, guidance/wellbeing officer, female] |
|  |  |  | *“I think it is overall because while there are some kids that don’t have phones, most of them do and most of them would rather engage with something like that using an app rather than, like if you gave them a diary or something.”* [Interview, year advisor/head teacher, female] |
|  |  |  | *…the feedback from students about the app indicated that it was a bit outdated and not engaging.* [Survey short answer, school counsellor/psychologist, female] |
|  |  |  | *“…there is probably barriers in staff kind of attitude to it… to the use of phones and things like that and I guess being you know… how schools in general see the use of phones and you know… are they allowed? Are they not allowed?”* [Interview, year advisor/head teacher, male] |
| *3. Ways of working within schools* | | | |
| *i. School buy-in and staff*  *ownership* | Process, individual characteristics: planning and engaging, knowledge of the intervention | School buy-in and ownership was dependent on a myriad of internal and external factors. Leadership support was necessary but not sufficient. Reputation of the implementing organisation and communication channels established between that organisation and schools are also important to encourage buy-in and knowledge about SPARX. | *“Yeah, I mean I did think that the principal might not get back actually, to be honest. Just because you know, another thing to, um, to do. And I was wondering whether he might be concerned about how much work might be required of teachers for the benefits of it, but I was really pleased when he approved it.”* [Interview, school counsellor/psychologist, female] |
|  |  |  | *“…a lot of support but not a lot of time to implement stuff, ahaha (sic)…so yeah, really requires individual’s taking on different projects and really running it themselves…”* [Interview, school counsellor/psychologist, female] |
|  |  |  | *“I just tend to go for black dog institute because the training that I did was pretty damn good [oh, good to hear, glad to hear]. All evidence-based and you guys work closely with the department…”* [Interview, school counsellor/psychologist, male] |
|  |  |  | *“Well I think if it was rolled out as a program in the school, I understand this is the study part…but if it was an established program rolled out to all students then I think it probably would be helpful for me at that point to know more about it.”* [Interview, school counsellor/psychologist, female] |
|  |  |  | *“I don’t know very much about the app at all actually I still don’t much about the app at all if I’m being honest with you…”* [Interview, school counsellor/psychologist, female] |
| *ii. Bringing all the right*  *players on board* | Process, inner setting: planning and engaging, individual role and capacity | There was staff consensus that involving the right people – those with the capacity, expertise, and interest/motivation to support student mental health or who have responsibility for the year group – at the right time is critical for effective implementation. Counsellors/psychologists strongly advocated to be key decision-makers about which programs are offered in their schools. | *“I would have preferred to be kind of involved from the start, I think it’s a decision that the school counselling service needs to be in on if that school has kind of capacity to kind of, not that ours doesn’t, being involved in that decision to see if we can do that, if it’s viable for our school…I think it’s really important that the school counsellor has a say in, before the before the survey is kind of taken on board I guess.”* [Interview, school counsellor/psychologist, female] |
|  |  |  | *“…the school counselling service needs to be a key person in all the communications and should have to agree to the dates of the study prior to those dates being set.”* [Survey short answer, school counsellor/psychologist, female] |
|  |  |  | *“I think that was a relevant part of what I do. And some of those kids aren't kids that, that I work with all the time. And it's, I'm happy to have exposure. So people know more about what I'm there to help do.”* [Interview, school counsellor/psychologist, male] |
|  |  |  | *I’m not going to lie… it had a big impact on my workload…for me to do that, there is a lot of paperwork involved in running any type of variation to routine [mmm] in a school, let alone informing the community about it as well and then physically doing it, it umm… it also takes away from lesson time… it disrupts umm… teachers from their classes coz they need to be supervising the program, so there’s a lot [mmm] that goes on behind the scenes, that maybe organisations aren’t aware of…”* [Interview, year advisor/head teacher, female] |
|  |  |  | *“…because our other school counsellor was on sick leave um but we do have two allocated to the school…but we weren’t getting our full allocation at that time so it was kind of just me doing three days on a five day load.”* [Interview, school counsellor/psychologist, female] |
| *iii. Communication &*  *support* | Process, inner setting: planning and engaging, individual role and capacity, networks, communication and support | Communication and support within the staff delivery team was described as helpful for planning and engaging students (e.g., gaining consent, organising sessions etc) and executing the visits, although it was not always present in some schools. | *“…another factor that played into this for us, was that we had a change of year advisors…* *So, we had one go on maternity leave... and because the information had come out at the… whether it was the very beginning of the year and I’d fed all the information and had a meeting with the Year Advisor about that [mmm] and then it all changed to be later in the year and then there was a different Year Advisor, it put stress on the Year Advisor, I know that. And they felt a little bit overwhelmed [mmhmm]… Umm I was helping them as much as I could, but you know, they’ve got to do their bit when it comes to organising things for their cohort [mmhmm]… and because they were new in the role, and had not done it before, I think that it just added that pressure.”* [Interview, year advisor/head teacher, female] |
|  |  |  | *I also feel that there is not enough communication between members of staff for this to be appropriate for our school at this point i.e. the counselling service was not even told when the surveys would be.* [Survey short answer, school counsellor/psychologist, female] |
|  |  |  | *“…to be honest with you I didn’t know about the Future Proofing study and that my involvement was, I was going to be involved until the first survey and then um I was contacted by the Head Teacher Well-Being saying that the old counsellor had forwarded her some emails um saying that kids needed to be checked in with…”* [Interview, school counsellor/psychologist, female] |
|  |  |  | *Worked well - great year advisor and school psychologist communication about who had consented, who to follow up on, logistics of booked rooms.* [Survey short answer, school counsellor/psychologist, female] |
|  |  |  | *“…challenging at times when, when you sort of delegate certain things and they might not get done [mm. Yep]... So that, that was tricky. So, it would’ve been better if I was here five days a week and I could, you know, oversee the running of it properly.”* [Interview, year advisor/head teacher, female] |
|  |  |  | *“I think that there wasn’t a lot of understanding about the time that those risk assessments take for us to do and fitting in with our other caseloads that we do needs to kind of blackout one or two full days to be able to do that well. That’s what I had to do at my school um and they weren’t always you know um super transparent with communications with us around what those dates would be, the uh surveys would be happening on which uh can be pretty logistically challenging in a school when I’ve got to be kind of be free to deal with those risk assessments, suicides are a kind of priority.”* [Interview, school counsellor/psychologist, female] |
| *iv. School visits and SPARX-*  *R sessions* | Process: executing, quality of support materials | School visits were generally described as being executed effectively whether delivered online or in person. | *“…the initial logging on was a little bit problematic as it is, anytime you're getting 30 kids to log onto a computer, in fact it was a lot less chaotic than I was expecting it to be. There were kids that didn’t know, ‘I don't have a password’ , ‘I don't have an email address’…”* [Interview, school counsellor/psychologist, male] |
|  |  |  | *“The people who were administering the zoom thing, that was a pretty simple, they just introduced themselves, brief rundown on what it was and off you go, if you need to ask any questions come up to the microphone off the air. And that was it was all pretty straightforward…”* [Interview, school counsellor/psychologist, male] |
|  |  |  | *“…trying to delegate tasks to other people, um, just in terms of having kids there on time for the session…ahaha (sic) [mm], that never went very smoothly, so and again, I wasn’t here to plan ahead [uhih] so, um, you know having the students know when and where to go for this survey, being prepared, was never done smoothly, so that was all very last minute. I had to quickly run around and get them out of classes and…just a bit of that planning stuff [uhih] wasn’t done very well. Um… and then obviously, we’re all adjusting to using technology [uhih], and, even though there were like… glitches, things still ran smoothly um because you guys provided us with enough information to just sort of run it ourselves.”* [Interview, school counsellor/psychologist, female] |
|  |  |  | *“…you want to schedule time for the kids to use the apps, you’d almost want the apps to be so engaging that you can use it in their own time organically… you know like “I want to keep playing… I want to keep learning…”* [Interview, head teacher/year advisor, male] |
|  |  |  | *“…some of the kids were just finding it difficult to find some time…. to [uhih] complete it. Um, I know that I think the school was encouraged to provide some time [uhih] for them to use the app but just in the busyness of high school day, [mm] um, particularly this year, with things being kind of shoved into only so many hours, that was little bit tricky to do.”* [Interview, school counsellor/psychologist, female] |
| *v. Screening and risk*  *alert system* | Process: executing | Identifying high-risk students through screening reportedly increased demand on counsellors/psychologists when appropriate provisions and planning was not in place to managed increased case load. | *“…the timing unfortunately which I may’ve mentioned in the survey [Yes] was um… just little bit difficult because Year 8s go off two periods, um, on those Mondays to sport. So, I can’t, I can’t see any of them when they are off site so it only left me with one period on that day to see them. And then the next day we had, I think I had six… or possibly seven students flagged so I had to enlist a colleague to help follow up. Simply the way works with the high school is that I phone the admin office. I ask or say that I need to see a student. They send the student up with the runner-go and ask the student to come down to the office, there’s no mention that it’s to see the counsellor, it’s just to come to the office. When they get to the office they are asked to go to the counsellor’s office and come to see me [Uhih]. Um, and um… yeah and then so I… usually I ask them if they understood why they were there. Because I wanted to get an idea of whether they were aware that they would be followed up [Uhih]. Um… If they were not aware then I simply explained and then did the risk assessment and worked out what follow-up they were gonna (sic) need…I think that worked well. Um, from my point of view, it was really timely, apart from, you know, the timetable clash, um, it, it worked well. Um, I think it was very useful.”* [Interview, school counsellor/psychologist, female] |
|  |  |  | *“…the first time that it happened I kind of had to drop everything um and cancel any other appointments that I had with kids to make sure that I could do justice to those risk assessments you know. Some kids’ risk assessments can take quite a few hours to set up with their parents and make sure that they are safe and things like that as well um so the first time it happens I did just have to kind of drop everything um to be able to do that. I then spoke to kind of my senior psychologist about it and about the gravity of the workload and we decided I would have to as I said to block out those dates to make sure that I did have the time to follow up those risk assessments that were so important but unfortunately the second survey, the school, I am not sure the communication was there, and the same thing happened again.”* [Interview, school counsellor/psychologist, female] |
|  |  |  | *The work load expected of the school counselling team is excessive and means that nothing else can be done for at least 2 days. It would be easier to manage if black dog would provide counsellors to complete some of the risk assessments on the day with the School counsellor/psychologist as part of the team. 9 risk assessments in 2 days with all the follow up that is needed is not feasible for a school psychologist or counsellor in the given time frame with the size of case load and work load of the counselling service currently, especially when no notice is given to the counselling service.* [Survey short answer, school counsellor/psychologist, female] |
|  |  |  | *School resources are too limited to supply adequate follow-up for students identified as being at risk.* [Survey short answer, school counsellor/psychologist, male] |
|  |  |  | *Identification and support of students in need worked well.* [Survey short answer, school counsellor/psychologist, Principal/deputy] |
| *4. Reflecting on past experiences to improve future implementation*  . | | | |
| *i. Future adoption* | Innovation characteristics, inner setting: relative advantage, relative priority, tension for change, appropriateness/acceptability | Reasons for future adoption as well as limits of the approach. | *“Destigmatises mental health, provides information to students about mental health, identifies potentially vulnerable students not known to the school counsellor. However, the feedback from students about the app indicated that it was a bit outdated and not engaging.”* [Survey short answer, school counsellor/psychologist, female] |
|  |  |  | *“it’s proactive, captures all um, helps students with the referral process, um, helps me develop, you know at least a face, because you know I, often I don’t have contact time with you know, year groups at a time, very much I am just a passing face so there is a selection of community who knows me really well [uhih] because they see me but then for most people, they kind of might’ve seen me walking around playing ground every now on then. They’ve never spoken to me so it just helps build the relationship as well [uhih] like a PR campaign”* [Interview, school counsellor/psychologist, male] |
|  |  |  | *Not straight away. If it proved to be successful I would definitely look at introducing it to Year 8 in the future. There is however, a lot of time involved in implementing the program and getting buy in from students and parents. There is also disruption to normal lessons and all logistical considerations that need to be taken into account.* [Survey short answer, year advisor/head teacher, female] |
|  |  |  | *I think the approach of the program was positive in nature and would be able to engage students in the ideas of mental health in a different way. I think with greater staff understanding and planning this would be able to be integrated into the school year and therefore achieve it's directed goals.* [Survey short answer, year advisor/head teacher, male] |
| *ii. Ideal implementation* | Process: planning and engaging, executing | Process factors that, according to staff, would facilitate optimal buy-in, engagement, and sustainability, overcoming barriers relating to resources, capacity, and coverage of students. | *“…it was able to be made as a part of the curriculum, like um…a particular unit that they are working on which is…um, often done in PDHPE where they were working on mental health specific sort of stuff. If that was sort of written into their program then that’s kind of more compulsory and all these things have to be done [mmmm, yep]. It’s not an additional, bit of work that people need to organise…”* [Interview, school counsellor/psychologist, female] |
|  |  |  | *I think if we could kind of fit this into a wellbeing day when all the kids are already off class, and the focus is all about wellbeing then it takes little bit of that, sort of the systemic pressure off.”* [Interview, school counsellor/psychologist, female] |
|  |  |  | *“I think the designation of class time would be very effective but I think it’s also got to go almost like a two or three pronged approach where the future proofing team would come in and actually educate the staff about it... because then that way it’s like… oh all the teachers are aware of what’s happening … there’s like a lot of moving parts to it, so I think using an onboarding process for the entire staff… even the ones who aren’t involved [mmhmm] would be a good way of doing that and it could be a good way of sort of tackling you know… the misconceptions of mental health as well... so it’s like… it’s kind of that… the idea is that you’re tackling the actual program itself but also educating adults about youth and young people and their mental health and sort of where they’re at and why this program is there.”* [Interview, year advisor/head teacher, male] |
